# Sensory Phenomena in Megrim

**Published:** 1900-02

**Authors:** J. C. Clemesha

**Affiliations:** M. D., M.R.C.S., Eng.; L.R.C.P., Lond., Buffalo, N. Y.


### SENSORY PHENOMENA IN MEGRIM.

By J. C. CLEMESHA, M. D., M.R.C.S., Eng.; L.R.C.P., Lond., Buffalo, N. Y.

MEGRIM, the English equivalent of the French migraine, is also known as sickheadache, blindheadache and some other terms and each of these are properly applicable to only one of the various forms that it may assume. Not in all forms of megrim is there disorder of sight or other sensory phenomena. In simple hemicrania we have periodical attacks of headache coming on, the pain being of a one-sided nature and nausea supervening only in severe attacks.

In the very common malady, familiarly known as sickheadache, the pain is localised in the forehead or other part of the head and is followed and relieved by vomiting. Usually the person is afflicted with a headache, coming on during the day, becoming more intense and later accompanied by a sense of nausea. Night commonly ends the attack. Sleep ensues, and the patient wakes next morning feeling as well as usual.

The next form of megrim we shall mention is a variety sometimes known as "blind headache" from the remarkable obscuration of vision which attends it. The disorder of sight may be present without the headache or nausea following. The headache may be on one or both sides and the blindness may occupy one half or the whole visual field.

The following is a description given me by A. B., at the Buffalo Eye and Ear Infirmary :

She is 22 years of age, and has been afflicted several years, at more or less regular intervals, and an attack can be brought on by attendance at a concert or the theatre, looking into store windows, and the like. She notices a cloudiness in front of the eyes beginning in the center of the field and gradually extending to the periphery, when the center becomes again clear. As the cloudiness extends it is bounded by a zigzag line of various colors which also gradually expands taking a horse-shoe outline. Within this zigzag line of colors is the blind area full of movements, as of boiling fluid and within this is the recovered area of vision. After lasting from ten to twenty minutes a severe headache follows, terminating some hours later by vomiting. In the case of another young woman, there was merely the transient cloudiness without the spectral phenomena.

Some patients have a transient hemianopia, as in the case of C. L., who could only see half an object when one of her headaches were coming on. In her case there was no prismatic effects though headache and vomiting followed. In the case of A. H. there was transient hemianopia in the left eye with transient loss of vision in the right, also numbness of the tongue and side of body, but no vomiting following the headache.

In more severe forms, though more rarely, other sensory disturbances occur. Tingling sensations, numbness in the face, limbs, tongue and other parts. These sensations are mostly unilateral and may be followed by transient weakness. Ptosis and a transient paralysis of the recti muscles have followed—aphasia may occur, words are forgotten or misplaced. Aphasia is commonly associated with a tingling on the right side. There may be mental depression, fear of impending evil or languor. All these sensory symptoms may be present without disturbance of vision, but more frequently follow the visual disorder in course of half an hour or so.

In speaking of the disorder of sight in megrim, I am reminded of the many patients who have visited the Buffalo Eye and Ear Infirmary on account of these sensory phenomena, having been alarmed with the idea that serious results might follow; also that many general practitioners do not seem to recognise these sensory symptoms as being a part only of a disorder and not due to a disease of the eyes. Whenever disorder of sight is present, it is an initial symptom, the first step in a series of sensory phenomena of which the paroxysm consists. It is a very common feature in severe cases being only secondary to the headache itself.

Dr. Liveing in his book on Megrim and Sick-headache states that we have the affection of the sight under two characters: (1) partial obliteration of the field of vision; (2) the spectral phenomena. He goes on to say with respect to the partial loss of sight that this may be central or eccentric. Usually, however, the patient first notices that he is unable to see what is directly in the center of the field of vision, or sees that the center is occupied by a bright spot.

There is a difference of opinion as to the degree of luminosity which the blank spot exhibits in various individuals; some call it white, some shaded, and some refer to it as a cloud, a general dimness or mist.

As to the other type, where the loss is other than central, it usually takes the form of loss of one half the field, usually the right or left half, more rarely the upper or lower half. In the same person the defect in the visual field may be sometimes central and at others lateral. This blindness is not stationary but progressive, extending in the central form from the center to the periphery while the sight gradually returns in the parts first affected.

One interesting case was seen some months ago, not so much as regards the blindness which was central with no spectral phenomena, but to a concomitant nervous affection which periodically troubled her. This patient, a woman of 38, at various times and most usually when lying down, in bed or otherwise, would find herself unable to rise and also unable to speak, still remaining quite conscious and able to discern any one coming into the apartment but could not move, hand, foot or tongue, remaining helpless until the temporary paralysis passed away, which it would do in the course of ten minutes or so. She had been this way for about seven years and could assign no probable cause, hereditary or acquired. This case seems somewhat analogous to a case of Thomsen's disease that I reported in the BUFFALO MEDICAL JOURNAL some years ago.

The spectral appearances vary in different people, from a faintly luminous border surrounding the blind area and attracting but little notice to highly defined and sharply pronounced bright colors, which remain vividly impressed upon the mind. Inside this luminous border is a rapid boiling, bubbling or glimmering motion, and sometimes when the appearances are more brilliant they look like showers of sparks. In regard to this spectrum it has never a well-defined outline, but seems to be made up of angles or zigzag lines.

Dr. Fothergill, who wrote many years ago, in a passage on Remarks on Sick Headache, says: "It begins with a singular kind of glimmering in the sight. Objects swiftly change their apparent position, surrounded with luminous angles like those of a fortification." It must be noted that the headache may be preceded by the blindness alone or the spectral appearances alone, though the both together is the usual sequence of events.

Ophthalmoscopic appearances do not throw any light upon the nature of these phenomena. Patients have had ophthalmoscopical examinations made in the various stages of the paroxysm, but the result of careful observations are negative.

As to the ocular symptoms the pupil is sometimes altered in size. Some observers state that it is always dilated, others that it is always contracted. The eyeball is said to be contracted in some cases and paralysis of the recti and ptosis sometimes result. The disturbance which causes the affection of sight must occur somewhere above the optic thalmus, as was long ago pointed out by Wollaston.

Many therories have been promulgated. These I shall not mention. In any case it is certain that no anatomical change is present as a cause of the disease.

329 FRANKLIN STREET.

